# On-farm dynamic management of genetic diversity: the impact of seed diffusions and seed saving practices on a population-variety of bread wheat

**DOI:** 10.1111/j.1752-4571.2012.00257.x

**Published:** 2012-12

**Authors:** Mathieu Thomas, Elise Demeulenaere, Julie C Dawson, Abdul Rehman Khan, Nathalie Galic, Sophie Jouanne-Pin, Carine Remoue, Christophe Bonneuil, Isabelle Goldringer

**Affiliations:** 1 INRA, UMR 0320 / UMR 8120 Génétique VégétaleGif-sur-Yvette, France; 2 CNRS, UMR Eco-anthropologie et EthnobiologieParis, France; 3 CNRS, UMR Centre Koyré d’Histoire des Sciences et des TechniquesParis, France

**Keywords:** *in situ* conservation, interdisciplinary study, network theory, seed exchange network

## Abstract

Since the domestication of crop species, humans have derived specific varieties for particular uses and shaped the genetic diversity of these varieties. Here, using an interdisciplinary approach combining ethnobotany and population genetics, we document the within-variety genetic structure of a population-variety of bread wheat (*Triticum aestivum* L.) in relation to farmers’ practices to decipher their contribution to crop species evolution. Using 19 microsatellites markers, we conducted two complementary graph theory-based methods to analyze population structure and gene flow among 19 sub-populations of a single population-variety [Rouge de Bordeaux (RDB)]. The ethnobotany approach allowed us to determine the RDB history including diffusion and reproduction events. We found that the complex genetic structure among the RDB sub-populations is highly consistent with the structure of the seed diffusion and reproduction network drawn based on the ethnobotanical study. This structure highlighted the key role of the farmer-led seed diffusion through founder effects, selection and genetic drift because of human practices. An important result is that the genetic diversity conserved on farm is complementary to that found in the genebank indicating that both systems are required for a more efficient crop diversity conservation.

## Introduction

Ten thousand years ago, human societies began to domesticate wild species so they could be easily cultivated, more productive, and better adapted to their needs (Diamond 2002). As the result of interactions between the environment, human uses and farming practices, these cultivated species were submitted to strong bottlenecks through genetic drift and artificial selection (Purugganan and Fuller 2009). This dynamic led to genetic differentiation in time and space, particularly at the molecular level, as shown by different levels of diversity between species and varying degrees of genetic structure, indicating a complex history (Haudry et al. 2007). The genetic diversity and structure of crops are typically studied at different scales in space ranging from the village level, which allows the characterization of diversity maintained by local community (Pressoir and Berthaud 2003), to larger regional distributions, which allow inferences about the evolutionary history of this species (Matsuoka et al. 2002; Delêtre et al. 2011).

Both farming communities and the scientific literature usually identify different varieties for a given cultivated species. Specific varieties within a species have been selected and used for a particular purpose and are distinct from other varieties of the same species by morphological traits and their particular use or quality characteristics. In contrast, diversity among individual plants within the variety so defined, (within-variety diversity), has rarely been characterized (Zhang et al. 2006). However, this component of the overall genetic diversity of a cultivated species is particularly sensitive to recent changes in farming practices. Modern methods of plant breeding, with the development of pure lines, caused a drastic reduction of the within-variety genetic diversity present in farming systems before the industrialization of agricultural systems (Roussel et al. 2004; Thomas et al. 2011). In addition, seed diffusion became linear and top-down from the plant breeder to the seed company and then to the farmer, and farmers purchased seed each year, stopping the adaptation process that occurs when farmers save and replant seeds of genetically diverse population-varieties (Bonneuil 2008).

In traditional farming, human and natural processes still strongly interact to determine the rate of change in population-varieties (Dyer and Taylor 2008). Two levels of human processes should be taken into account: first, the seed diffusion between farmers; second, cultural practices, including selection (also termed ‘artificial selection’ to distinguish it from ‘natural selection’), and seed storage conditions. Because farmers use their own saved seed for several years, seed diffusions are not very frequent (Perales et al. 2003). Farmers’ selection is generally applied on inflorescences (ears or panicles), which may induce kin-structured founder effects, as seeds in a single inflorescence are full or half-sibs. This kin-structured founder effect can cause an increase in differentiation among populations (Louette et al. 1997; Ingvarsson and Giles 1999). Environmental processes also include stochastic events such as catastrophic weather (strong drought, flood...). Thus, an extinction event can be the result of a climatic disaster or of a farmer’s decision not to grow a particular variety (sub-population) in a particular field and year. Local extinction occurs when a seed lot is not re-sown for various reasons. Colonization occurs when a new population arrives in a new farm after a diffusion event between two farmers. Farmers generally receive seed from a single source (propagule pool-like situation) (Rice et al. 1998) or from a limited number of sources (Almekinders et al. 1994; Zeven 1999; Perales et al. 2003; Alvarez et al. 2005; Badstue et al. 2007; Hodgkin et al. 2007; Barnaud et al. 2008).

In industrialized countries, although landraces and folk varieties are no longer cultivated by the majority of farmers, seed saving and seed exchange networks have recently emerged in the context of organic agriculture [reviewed by Thomas et al. (2011)]. Organic farmers, faced with a shortage of varieties meeting their needs in terms of agronomic and quality traits, have begun cultivating varieties obtained from genebanks or from elders. Farmers within these associations generally exchange small quantities of seed which are then multiplied on farm for their own use. While these seed exchanges share characteristics with the informal seed systems of traditional agricultures, they also have specificities as they are situated in the context of modern organic agriculture in developed countries (recent social connection among farmers through seed circulation, renewing of communities of practices, long-scale seed exchanges, etc.…) (Demeulenaere and Bonneuil 2012).

The role of this type of seed exchange network in the conservation of genetic diversity in an industrialized context can be important but is not yet well characterized. In this paper, we develop an interdisciplinary approach by combining genetics and ethnobotany to assess for the first time the level of genetic diversity and the population structure at the variety level, from the example of Rouge de Bordeaux (RDB), a folk variety of bread wheat distributed among a network of actors in France. Our goal was to assess to what extent seed diffusion and farming practices influence the genetic diversity of this variety and its population structure. Outcomes from this research could contribute to the proposition of recommendations in terms of management strategies of crop diversity.

## Materials and methods

### Population origin and sampling strategy

Initially, a socio-anthropological study focused on the dynamics of seed circulation within the social network composed of farmers from the national Réseau Semences Paysannes organization, an organization created in 2003 to revive on-farm management of seeds and linking concerned farmers’ associations (literally ‘Peasant seed network’, below referred to as RSP) and with the curator of the French National Genebank at Clermont-Ferrand (CLM). A snowball approach was used to trace back seed circulation of bread wheat varieties among the different actors. This study revealed that RDB was one of the most popular varieties among farmers in the RSP (Bonneuil and Demeulenaere 2007).

Historical archives revealed that RDB appeared probably around 1865 in Lectoure, in the south-western France, then started moving toward Bordeaux (still in south-western France) and toward the central France during the years 1870–1871 (Vilmorin-Andrieux Companie 1880). RDB was present in at least 75% of French departments in 1912 (Brétignière 1912). Afterward, its use began to decline as it was replaced by more productive varieties. Wheat varieties of the time were mostly genetically heterogeneous. For this reason, they are called population-varieties, following Bustarret’s definition (1944). RDB is thus a population-variety characterized by its ear type, which is red and awnless.

**Figure 1:**
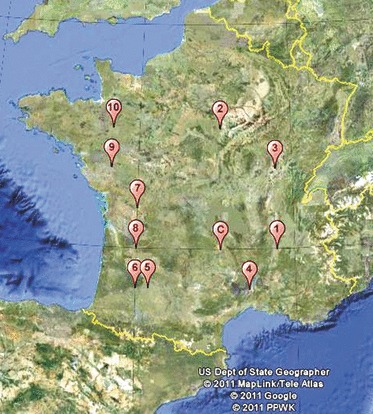
Location of the 10 actors growing Rouge de Bordeaux (RDB) populations in France (1–10), plus the location of the national genebank (C).

Relying on this information, we asked the genebank curator and some farmers cultivating RDB to provide us one or more seed samples from their populations. The nomenclature used to identify each sample was as follows: the first three-first characters for the name of the seed lot provider and two characters for the year of the last harvest. One optional letter was added if two samples came from different seed management practices on the same farm in the same year. We obtained 19 seed samples from 11 actors distributed among the French territory (for the privacy of the farmers, we have used code names) (Table 1, Fig. 1).

**Table 1:** Summary description of the 19 sampled populations

					Coordinates			
						
Seed sample name	Location	Receipt year	Sampling year	No. of reproduction cycles	Longitude	Latitude	Altitude	Population size
ALP05	1	1991	2005	14	5.815	45.154	588	Large
ALB06B	2	1998	2006	8	3.814	48.621	78	Medium
ALB06C	2	1998	2006	8	3.814	48.621	78	Medium
ALB03A	2	1998	2003	5	3.814	48.621	78	Small
ALB03B	2	1998	2003	5	3.814	48.621	78	Medium
BER03	3	1999	2003	4	5.270	47.561	296	Small
BER06	3	1999	2006	7	5.270	47.561	296	Small
JEF06	4	1998	2006	8	4.506	44.093	225	Large
JFB03	5	1998	2003	5	0.426	44.255	64	Large
JFB06	5	1998	2006	8	0.426	44.255	64	Large
JFB05	5	1998	2005	7	0.426	44.255	64	Medium
PHC06	6	2000	2006	6	0.526	44.354	86	Medium
FRP06	7	2005	2006	1	0.666	46.154	33	Medium
JOP06	8	2004	2006	2	0.221	45.843	97	Medium
VIC06A	9	2005	2006	1	1.133	47.012	90	Medium
VIC06B	9	2004	2006	2	1.133	47.012	90	Medium
JAS04	10	2003	2004	1	4.506	44.093	225	Medium
CLM03	C	1984	2003	11	3.143	45.775	336	Small
CLM04	C	2003	2004	1	3.143	45.775	336	Small

Seed sample name: the three-first characters represent the seed lot provider, two numerals for the year of the last harvest and one optional characters was added if more than one sample was provided by the same farmer the same year; Location corresponds to the number used in Fig. 1 to localize the origin of the seed samples; Receipt year: year of the last diffusion (colonization) event; Harvest year: year of the last harvest of the seed sample; No. of reproduction cycles: number of reproduction cycles from the last diffusion event; Coordinates: geolocalization data of the seed samples; Population size: qualitative population size of the sampled populations based on the cultivated area (small = 1–10 m^2^, medium = 10–100 m^2^, large > 100 m^2^).

Interviews focusing specifically on sampled populations of RDB were performed to obtain more detailed information about seed circulation and cultural practices. Applying the snowball approach to trace back the seed circulation of RDB, new actors mentioned during the interviews were contacted and interviewed. For each dissemination event, we recorded the actors involved, the date, and when this information was available, the quantity of seed diffused.

Although farmers involved in seed systems have received increasing attention as potential partners for participatory plant breeding and development programs (McGuire 2008), only few studies depict these systems through an analysis and the graphic representation of seed exchange networks (Subedi et al. 2004; Bonneuil et al. 2006; Aw-Hassan et al. 2008; Emperaire et al. 2008). In these studies, seed exchange networks between farmers were drawn in which the node corresponds to the farmer and the link materializes the seed flow. Depending upon the study, a multi-species or multi-variety seed exchange network was represented. In this study, to better understand the consequences of actor practices on the genetic structure of the crop, we focused on the partial seed diffusion and reproduction (number of generations) networks at the population-variety level (RDB). In our case, the node corresponds to the wheat population seed lot and the link combines the seed flow and reproduction.

### Molecular analyses

In the spring of 2007, leaf samples were taken from 13 to 44 plants per population (mean number of plants: 31), sown on November 8, 2006 at Le Moulon experimental station. For each plant, total DNA was extracted from 50 mg of fresh material following a protocol derived from the Dneasy 96 Plant Kit (QIAGEN, Valencia, CA, USA). Sixteen microsatellite markers developed by Röder et al. (1998): Xgwm135, Xgwm149, Xgwm161, Xgwm234, Xgwm257, Xgwm260, Xgwm272, Xgwm372, Xgwm400, Xgwm413, Xgwm415, Xgwm437, Xgwm469, Xgwm480, Xgwm539, and Xgwm642, one (wmc231) by Somers et al. (2004), and a bi-loci marker (CFD17) on two chromosomes by Guyomarc’h et al. (2002) were used for genotyping the 586 individuals studied. This set of 19 markers covers 19 out of the 21 chromosomes of bread wheat. Only chromosomes 1A and 6B were not covered. PCR protocols were adapted from Röder et al. (1998) and Guyomarc’h et al. (2002): an initial denaturation (3 min at 94°C), and 35 cycles of 30 s at 94°C for denaturation, 30 s at 50°C (between 45 and 60°C, depending on the primer) for annealing and 30 s at 72°C for extension, followed by a final extension step of 5 min at 72°C. Amplified fragments were separated on a ABI 3130xl semi-automatic sequencer (Applied Biosystems, Courtaboeuf, France) and analyzed with GeneMapper 3.7 (Applied Biosystems, Courtaboeuf, France).

Flowering time is a major adaptive trait in plants and in particular in the case of wheat because it determines the environmental conditions of reproduction with respect to climate and pathogen pressures (Remington and Purugganan 2003; Goldringer et al. 2006; Rhoné et al. 2008; Rhoné et al. 2010). The VRN-1 gene has been shown to be strongly associated with flowering time in wheat (Yan et al. 2003, 2004; Rhoné et al. 2010; Rousset et al. 2011). In addition, wheat experimental populations cultivated for several years in either northern or southern France have shown significant contrasting responses in terms of allele and haplotype frequency variation (Rhoné et al. 2008; Rhoné et al. 2010). Thus, to search for some adaptation to climatic conditions in the populations, four VRN-1 polymorphic sites located in the three orthologous copies of VRN1 were genotyped: (i) duplication, insertion, and deletion in the promoter of VRN-1A (denoted VRN-1Apr in the following) revealed by Yan et al. (2004), (ii) a substitution in the seventh exon of VRN-1A (VRN-1Aex7) revealed by Sherman et al. (2004), (iii) a 4-kb deletion in the first intron of VRN-1B (VRN-1Bint1), and (iv) a 4-kb deletion in the first intron of VRN-1D (VRN-1Dint1) revealed by Fu et al. (2005). For all the VRN-1 polymorphic sites, PCR conditions and PCR product digestion protocols were the same as defined by the authors. To detect variations at VRN-1Apr, forward primers were modified with an M13 extension according to Boutin-Ganache et al. (2001), and PCR amplifications were performed in the presence of fluorescent-labeled M13 extension. The amplification products, loaded on 6.5% denaturing polyacrylamide gels, were analyzed on a LI-COR automated DNA sequencer (LI-COR Biosciences, Lincoln, Nebraska USA). The variations at VRN-1Aex7 (CAPS marker) and at VRN-1Bint1 and VRN-1Dint1 (presence or absence of deletions) were revealed by migration on 2% and 0.8% agarose gels, respectively, and visualized with UV light.

### Genetic analyses

Population structure was assessed at two levels, among and within populations.

#### Genetic structure among populations

The multivariate graph theory method Population Graphs developed by Dyer and Nason (2004) was used to study the genetic structure among populations. This approach is derived from graph theory and aims to describe complex population structures based on the distribution of the genetic covariance among the studied populations using SSR molecular data. Individuals of each population define a multidimensional population centroid. Each centroid defines a unique multidimensional coordinate representing the average genetic individual within the population considered. The same pairwise distances as in amova (Excoffier et al. 1992) were calculated, and a weighted saturated Population Graph was drawn where the weight corresponded to the distance. An informative topology was obtained by selecting an edge set that sufficiently described the among-population genetic covariance structure. Relying on genetic covariance properties and conditional independence, Whittaker (1990) proposed a statistical test to perform this edge selection with an alpha level for the fit of the network after edge removal set to 0.05. The network was constructed using the software GENETIC STUDIO (Dyer 2009). To quantify differentiation among sampled populations, we used the conditional graph distance metric (cGD), which is estimated as the length of the shortest path connecting pairs of populations, following Dyer et al. (2010). Values of *F*_ST_ were also estimated for each pair of populations using Weir and Cockram’s Θ estimator (Weir and Cockerham 1984) implemented in GENETICS software (Belkhir et al. 2000).

To understand the general organization of the Population Graph, it was necessary to detect whether structural sub-units (communities) were associated with more highly interconnected parts of the network. A deterministic approach that detects potentially overlapping communities based on the Clique Percolation Method with weight (CPMw) was performed using Palla’s algorithm implemented in CFinder software (Adamcsek et al. 2006). In this approach, a *k*-clique is defined as a complete subgraph of *k* nodes all linked together (*k*−1 edges per node). Then, a community corresponds to the union of all *k*-cliques that can be reached from one to the other through a set of adjacent *k*-cliques (where adjacent means share *k*−1 nodes). The inverse of the distance matrix was used as a weighted matrix for the community detection. Communities can then be defined using an algorithm adapted for the weighted networks (Farkas et al. 2007). The intensity threshold (*I*) and the size of the clique (*k*) need to be chosen to have the lowest possible values while avoiding the detection of a single giant network. No giant network appeared when *k* is equal to 3 and without a fixed threshold for *I*. The algorithm was therefore used with these parameters.

#### Within-population genetic structure

Genetic diversity was studied for both the 19 neutral markers and the four loci (VRN-1Apr, VRN-1Aex7, VRN-1Bint1 and VRN-1Dint1) located in three orthologous genes (VRN-1A, VRN-1B and VRN-1D). Mean number of alleles (*R*_S_), unbiased Nei’s estimate of genetic diversity (*H*_e_) (Nei 1978), mean observed heterozygosity (*H*_o_), and the deviation from Hardy–Weinberg genotypic proportions (*F*_IS_) were calculated with Genetix software (Belkhir et al. 2000). Genotype richness (also called polyclonality) was estimated as the number of unique genotypes divided by the number of individuals per population. Following Goldringer and Bataillon (2004), we estimated the effective population size (*N*_e_) using the temporal method proposed by Waples (1989) that relies on the variance of allelic frequency (*F*_c_): 
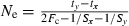
, where *S*_*x*_ is the number of individuals sampled at the *t*_*x*_ generation (respectively *S*_*y*_ individuals at *t*_*y*_).

The fine population structure was studied considering each genotype as two haplotypes. Haplotype reconstruction and inference of missing data were performed using PHASE software (Stephens et al. 2001). Based on the methods of a recent paper (Garrick et al. 2010), the MR algorithm was used. Runs consisted of 100 iterations as burn-in, 100 main iterations, and thinning interval equal to 1. Recombination rate between loci was equal to 0.5 because all markers were on different chromosomes. Then, pairs of haplotypes were selected using the best probability for each individual. This new dataset constituted a phased Multi-Locus Genotype (pMLG) dataset that was used with Arlequin software (Excoffier and Lischer 2010) to compute the inter-haplotype distance matrix, that is, the number of differences between each pair of haplotypes. We drew a saturated weighted network with each node corresponding to a distinct haplotype and edges linking each pair of haplotypes. Then, a threshold was fixed at one difference between haplotypes to conserve a link between two haplotypes. The haplotypic network was drawn with the Pajek software (Batagelj and Mrvar 2002). Kamada–Kawai’s force-based algorithm (Kamada and Kawai 1989) was used to provide spatial distribution of the unconnected sub-networks composed of sets of nodes connected together and further called connected components. Each connected component composed of more than two nodes was defined as an independent haplotype class. Other haplotypes were defined as off-types (OT). The Minimum Spanning Network (MSN) obtained with these haplotypes was also drawn. The network representation of this MSN was achieved with the Pajek software (Batagelj and Mrvar 2002) with each node corresponding to a distinct haplotype and one edge linking two haplotypes with one difference. Color of nodes corresponds to the haplotype class of each haplotype. Intermediate haplotypes that were not observed were represented by ‘.’ on haplotype networks. The same procedure was followed to determine haplotype frequencies and MSN for the four markers in the VRN1 gene copies, except that because no double heterozygote was found in the dataset, genotypes have not been phased.

Haplotype variation within populations was calculated by estimating the unbiased genetic diversity (*H*_d_), which accounts for small population sizes, computed as: 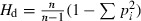, where *n* is the number of gene combinations analyzed in a population and *p* is the frequency of the *i*th haplotype in a population (Nei 1987).

A shared haplotype network (SHN) was drawn to track haplotypes represented at low frequencies among populations. Two populations were considered connected if they shared at least one haplotype. A threshold of haplotype occurrence in the whole dataset was set to 50 to represent only rare haplotypes. The Clique Percolation Method (CPM) was performed on the SHN using Palla’s algorithm implemented in Cfinder software (Adamcsek et al. 2006), to detect communities of populations characterized by their shared allele composition.

Student’s tests were performed using R software (R Development Core Team 2005) to test (i) whether populations taken in each of the seed diffusion and reproduction networks (SDRN, connected components) detected based on the interviews were more distant than populations from the same SDRN, (ii) for a significant difference between the mean values of diversity indexes estimated in each independent SDRN.

## Results

### Seed diffusion and reproduction of RDB populations

**Figure 2:**
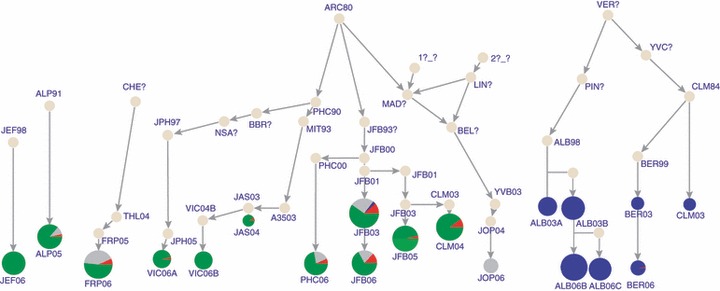
Seed diffusion and reproduction networks of the Rouge de Bordeaux (RDB) population-variety: nodes represent seed lots identified as follow: the three-first characters for the name of the seed lot provider, two numerals for the year of the last harvest and optional character if two samples came from the same farm in the same year. A question mark is used when information is not available. Seed diffusion events are represented in the time from the top to the bottom. Vertical arrows represent several cycles of reproduction on the same farm. Horizontal or slanting arrows represent seed diffusion events. Sampled populations are represented by pies of different colors corresponding to their composition in class of haplotype (Class I is composed of haplotypes in blue, Class II is composed of haplotypes in gray, class III is composed of haplotypes in green, class IV is composed of haplotypes in light green, class off-type is composed of haplotypes in red) details are provided in Fig. 5A.

The interviews with the different actors allowed us to trace the circulation of RDB populations to almost 30 years back. Thirty-five populations of RDB were documented with 28 seed diffusion events identified between 17 actors in addition to the 11 who provided seed samples. Populations were grown from 1 to 14 generations on the same farm. Based on this information, an oriented SDRN was drawn (this information was summarized in Fig. 2). Nodes represent seed lots of RDB and edges represent diffusion or reproduction events for these seed lots. This information defined two connected components (SDRN1 and SDRN2) where each node is a RDB population described by a location (farmer’s name), a year and an optional character for multiple samples from the same farm and in the same year (see Fig. 2 and Table 1 for details). VIC provided us with two samples from two origins (VIC06A and VIC06B). Among the 19 sampled RDB populations, seven were connected together in the first SDRN (SDRN1). They shared a common ancestral population maintained in the Vilmorin-Verneuil collection (VER?). This SDRN included the seed lot maintained by the French genebank (CLM03). A second connected component (SDRN2) was detected grouping nine other RDB populations. These populations shared a common ancestral population grown between 1980 and 1993 in an alternative community farm (ARC80). This population was alternatively cultivated within a mixture composed of at least three distinct varieties and as a pure variety after a selection step based on spike type. Incomplete information made it impossible to connect three populations (JEF06, FRP06, and ALP05) to any network. Our knowledge about seed diffusion thus does not extend back far enough in time to find a seed diffusion event that connected the two connected components.

The interviews with the different actors indicated that three main cultural practices were observed: populations grown on small (1–10 m^2^), medium (10–100 m^2^), or large (>100 m^2^) plots. These different areas corresponded to different functions: small plots were used for collections of several varieties (ALB03A, BER03, BER06, CLM03, and CLM04); medium plots are also used for collections of a few varieties or multiplication of seed lots to increase the seed quantity as preliminary step before production (ALB03B, ALB06B, ALB06C, JFB05, PHC06, FRP06, JOP06, VIC06A, VIC06B, and JAS04); and large plots corresponded to production in fields (ALP05, JEF06, JFB03, and JFB06). Practice diversity is observed among farms but also within farms. For example, ALB used three different practices on his farm. ALB03A corresponded to a population maintained in collection (small plot). ALB03B and ALB06B are temporal samples of the same population maintained following conservation practices (selection for a particular varietal phenotype), with seed samples grown on 10 m^2^ (medium). ALB06C has been grown in isolation within a field of another species (medium plot size). We also learned that JOP06 applied spike mass selection when he received the RDB in mixture with other varieties. JFB made a selection within his RDB population in 2001 based on an ear type with awns. This population was sampled in 2005 after four generations cultivated independently to his RDB population (JFB05). Another sample of this selection was obtained by CLM and was provided for this study after one cycle of reproduction using the conservation practices of CLM (CLM04).

### Allelic within-population diversity

The level of genetic diversity estimated in each population with the unbiased Nei’s index showed a large range of values (between 0.01 and 0.35, Table 2). An estimation of the effective size (*N*_e_) was possible for the only temporal samples we had: the JFB and BER populations between 2003 and 2006 (respectively JFB03–JFB06 and BER03–BER06). Genetic effective population size was estimated as 104.5 individuals for the JFB population. *N*_e_ tended toward infinite for the BER population because allele frequencies varied only very little leading to a very low *F*_c_ value compared with the sample size effect.

**Table 2:** Diversity indexes computed for all 19 populations based on 19 SSR markers. Seed diffusion and reproduction networks (SDRN) indicates from which seed diffusion and reproduction network each seed sample belongs according to Fig. 2. Genetic group indicates the genetic group assignation of each sample according to the results of the Fig. 3A

Seed sample name	Sample size	SDRN	Genetic group	*H*_e_	*H*_o_	*R*_S_	GS diversity	*H*_d_	Polyclonality	*F*_IS_
ALP05	29	Unknown	2	0.16	0.01	2.26	0.21	0.46	0.28	0.97
ALB06B	13	1	1	0.02	0.01	1.11	0.27	0.37	0.23	0.37
ALB06C	28	1	1	0.03	0.00	1.11	0.18	0.50	0.11	1.00
ALB03B	32	1	1	0.00	0.00	1.05	0.13	0.03	0.06	0.00
ALB03A	32	1	1	0.04	0.00	1.26	0.21	0.62	0.22	0.91
CLM03	32	1	1	0.00	0.00	1.05	0.06	0.06	0.06	1.00
CLM04	30	2	2	0.10	0.03	1.89	0.27	0.46	0.33	0.68
BER03	41	1	1	0.01	0.00	1.21	0.06	0.19	0.10	1.00
BER06	44	1	1	0.04	0.00	1.84	0.09	0.16	0.11	0.97
JEF06	31	Unknown	2	0.05	0.00	1.53	0.20	0.67	0.42	0.97
JFB03	31	2	2	0.32	0.01	2.53	0.31	0.90	0.52	0.97
JFB06	38	2	2	0.31	0.03	3.32	0.22	0.71	0.45	0.91
JFB05	29	2	2	0.19	0.02	2.21	0.25	0.77	0.59	0.87
PHC06	29	2	2	0.27	0.00	2.21	0.21	0.80	0.41	0.99
FRP06	29	Unknown	2	0.35	0.01	2.53	0.29	0.81	0.48	0.98
JOP06	29	2	2	0.01	0.00	1.11	0.08	0.17	0.14	0.79
VIC06A	29	2	2	0.05	0.00	1.68	0.12	0.37	0.24	1.00
VIC06B	30	2	2	0.04	0.00	1.42	0.12	0.57	0.33	0.96
JAS04	30	2	2	0.09	0.00	2.21	0.05	0.39	0.27	0.98

With *H*_e_: unbiased Nei’s estimate of genetic diversity (Nei 1978), *H*_o_: mean observed heterozygosity, *R*_S_: mean number of alleles, GS diversity: the multivariate genetic diversity index (Dyer and Nason 2004), *H*_d_: unbiased genetic diversity for haplotypes, *F*_IS_: the deviation from Hardy–Weinberg genotypic proportions.

### Structure of genetic diversity among populations

**Figure 3:**
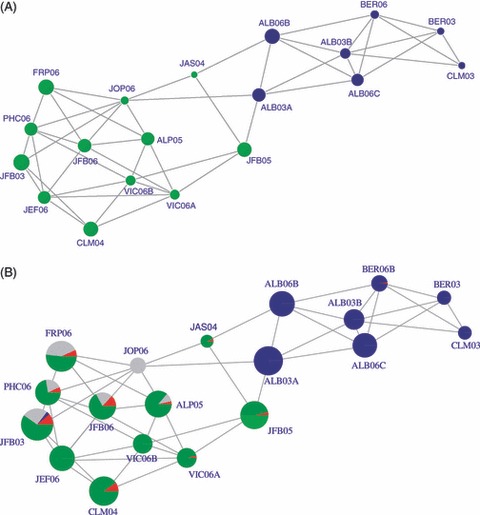
Population Graphs obtained with Genetic Studio software based on 19 SSR markers. (A) Community detection was performed with CFinder software with *k* = 3 (*k* = clique size) and without intensity threshold on the weight of edges. Two nonoverlapping communities were detected: one represented in blue color (seven populations), the other in green color (11 populations). A third overlapping community is represented with a gray circle. (B) Pies illustrate the population structure of each population based on the five classes of haplotypes (class I in blue, class II in light blue, class III in green, class IV in light green and class off-type in white) defined in Fig. 5A. The node size is proportional to the haplotype diversity of the population for both graphs.

Based on SSR molecular data and using the conditional independence method, the network topology that fits the global genetic covariance held in the dataset with an alpha error of 0.05 needed 47 edges to link the 19 RDB populations. This network clearly showed two groups of populations (group1 and group2) where populations from the same group were more connected than populations from different groups. This observation was confirmed by a community detection using CPMw algorithm. Two nonoverlapping communities were detected for a size *k* = 3, with *k* being the clique size parameter in the community search algorithm. The first one contained seven populations (denoted as group1) and the other 11 populations (denoted group2) (Fig. 3A; group1 in blue and group2 in green). A third overlapping community was also detected (JAS04, ALB06B, and ALB03A), making the link between the two nonoverlapping communities.

**Figure 4:**
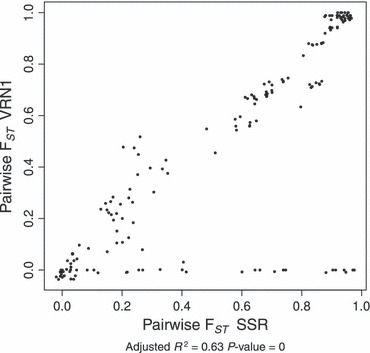
Biplot of between pairwise *F*_ST_ of 19 SSR markers and four VRN1 markers.

The Population Graph obtained for the four *VRN1* loci revealed a similar structure (data not shown). Eighteen among the 19 studied populations fell into the same groups regardless of the kind of marker. Only JOP06 was in the green group for the SSR markers but in the blue group for the VRN1 genes. This result was confirmed by a strong correlation between pairwise *F*_ST_ computed for SSR markers and VRN1 genes, respectively (Fig. 4). Points with a pairwise *F*_ST_ VRN1 value close to 0 and a pairwise *F*_ST_ SSR value above 0.5 corresponded to pairs of populations comprising JOP06 and one of the populations from group1.

### Haplotypic structure of RDB

#### Individual haplotypic structure

**Figure 5:**
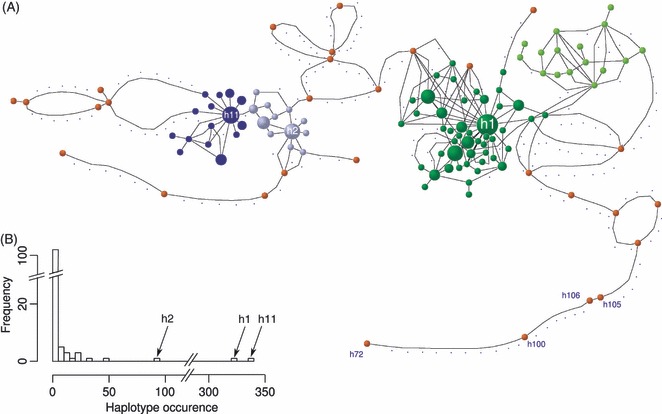
(A) Haplotype spanning network based on the 586 individuals (119 different haplotypes) in the dataset. The size of the node is proportional to the haplotype occurrence among the 19 populations (between 1 and 11). Class I is composed of haplotypes in blue, Class II is composed of haplotypes in gray, class III is composed of haplotypes in green, class IV is composed of haplotypes in light green, class off-type is composed of haplotypes in red). (B) Distribution of haplotype occurrence based on the 586 genotypes of the dataset.

The MSN based on the 19 SSR multilocus genotypes (MLG) included 119 distinct nodes, where each node was a distinct haplotype. The haplotype distribution among individuals (Fig. 5A,B) showed two main haplotypes (h1 and h11: 321 and 339 occurrences, respectively) differing at 12 of the 19 loci. A third haplotype (h2) was detected 91 times and was close to h11 (separated by four differences). These three haplotypes contributed for 64% of the whole dataset. The remaining haplotypes were detected from one to 47 times, and of these haplotypes, 76% were rare (i.e., present fewer than three times). The network topology of this MSN showed that most of the minor haplotypes were closely connected to the three main ones which suggested that they could be variants around the main haplotypes. The haplotype network, where two nodes were connected if the two haplotypes differ by one difference, showed four connected components composed of more than two nodes (Figure S1). Based on this property of the network topology, we defined four classes of haplotypes (Fig. 5A): class I included h11 and 14 closely connected haplotypes (in blue in Fig. 5A), class II included h1 and 45 close haplotypes (in green), class III included h2 and 11 close haplotypes (in gray), and we also defined as class IV (in light green), a set of 16 haplotypes found at a low frequencies but highly connected (differing at one or two loci). This class was closely connected to class II (Fig. 5A). Finally, 29 haplotypes were considered as OT because they were too distant from the four classes. Among them, haplotypes h100, h106, and h105 (observed in populations CLM04 and FRP06) seem to derive from recombination between another off-type (h72) and one of the main haplotypes (h11).

#### Within- and among-population haplotypic structure

Using the previous haplotype clustering, we plotted the frequency of each haplotype group in the sampled populations, using pie charts on the Population Graph presented in Fig. 3B. This representation confirmed the existence of two main genetic groups of sampled populations. Each group showed a distinct pattern. The first one (in blue) (BER03, BER06, ALB03A, ALB03B, ALB06B, ALB06C, CLM03) was clearly homogeneous and mainly composed of class I haplotypes with a majority of the h11 haplotype. The rest was satellite haplotypes bearing between 1 and 3 differences compared with the h11 haplotype. Very few OT (<1%) were observed in this group of populations. The second genetic group was mainly composed of haplotypes of class II. JAS04, one of the three overlapping populations between the two groups, presented the same pattern. Thus, it seems sensible to bring it closer to the second genetic group rather than to the first group. The same argument could be applied for ALB06B and ALB03A to move them closer to Group1. Group2 was clearly more heterogeneous. Some populations were composed of individuals bearing mainly haplotypes of class II (JEF06, CLM04, VIC06A, VIC06B, and JAS04), one population (JOP06) was composed of individuals bearing haplotypes from the unique class III, while the rest consisted in composite populations composed of individuals of class II and III haplotypes (PHC06, JFB06, ALP06, FRP06) except for the population JFB05, which included haplotypes from classes II and IV. Only one population (JFB03) had individuals that shared haplotypes from three classes (I, II, and III). The proportion of off-type haplotypes in this second genetic group was higher than the first genetic group, with on average 4% OT per population.

**Figure 6:**
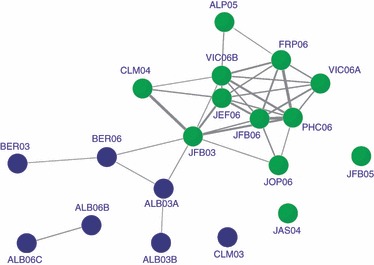
Shared haplotype network of Rouge de Bordeaux (RDB) sub-divided populations. Two nodes are linked if they shared at least one rare haplotype (present <50 times). The width of edges is proportional to the number of haplotypes (from 1 to 9). The color of the nodes corresponds to the genetic group detected in Fig. 3A (population samples from group1 are in blue, population samples from the genetic group2 are in green).

A SHN was drawn to track haplotypes that were present in different populations at low frequencies (Fig. 6). A 6-clique community composed of six populations was found (PHC06, FRP06, JFB06, JEF06, VIC06A, VIC06B). This finding highlights that a set of haplotypes is shared by several populations. The 5-clique community included JFB03 in the group of six populations. Two other populations (CLM04 and JOP06) were connected to this core in the 4-clique community. All of these populations had been previously assigned to group2. A 3-clique community was found composed of three populations (ALB03B, BER03, and JFB03). Owing to a class I haplotype shared with JFB03, this community overlapped with the 3-clique community comprised by the populations already included in the 4-clique community. This was because JFB03 shared a class I haplotype.

### Cross analysis between seed circulation information and genetic data

Based on our knowledge on seed diffusion, a pairwise matrix between the 16 populations belonging to a known diffusion and reproduction network (SDRN1 or SDRN2) was built to describe whether two populations belong to the same connected component or not. To quantify genetic differentiation among sampled populations, averaged cGD were computed within each group and between the two groups on the Population Graph. We tested for a significant difference in cGD values within and between groups using a Student’s test. The difference was highly significant (*P*-value < 2.2 × 10^−16^) with cGD averaging 5.8 for populations belonging to the same SDRN and 22.8 for populations that did not belong to the same SDRN. This result was consistent with the high level of differentiation observed between the two genetic groups detected in (Fig. 3A) (*F*_ST_ = 0.697). Comparison between mean diversity indexes for SDRN1 and SDRN2 shown **s**ignificant Student’s tests for *H*_e_ (*P*-value = 0.01 with *H*_e_ = 0.02 and *H*_e_ = 0.15, respectively), *R*_S_ (*P*-value = 0.005 with *R*_S_ = 1.23 and *R*_S_ = 2.06, respectively), *H*_d_ (*P*-value = 0.02 with *H*_d_ = 0.27 and *H*_d_ = 0.57, respectively), and Polyclonality (*P*-value < 0.001 with Polyclonality = 0.12 and Polyclonality = 0.36, respectively). Only GS diversity, *H*_o_ and *F*_IS_ were not significant (Table 2). This body of evidences indicated that the information on seed diffusion gathered through interviews was strongly consistent with the genetic structure detected with molecular data and that seed diffusion strongly influence the genetic structure and the levels of diversity of the managed populations.

Three populations were not assigned to any SDRN. JEF06 was composed of haplotypes from class III, and ALP06 and FRP06 were composed of haplotypes from classes II and III (Fig. 3B). These results suggested that they were closer to SDRN2 than to SDRN1. This finding was confirmed by the fact that JEF06 and FRP06 were included in the 5-clique community (Fig. 6).

## Discussion

### The RDB population structure

This study analyzed the structure of genetic diversity in a subdivided bread wheat population-variety named RDB. The sub-populations have been circulated for several years in a network of French actors (including farmers and the national genebank) involved in conservation and use of crop diversity. The goal of these analyses was to provide insights into the history of the populations to assess the impact of human practices on genetic diversity at the molecular level, to guide decisions on the conservation of genetic resources. In this study, we did not analyze quantitative genetic variation of adaptive or economical significance.

We applied the Population Graph method (Dyer and Nason 2004), which is a network theory-based method, to study inter-population relationships rather than *F*_ST_-based or distance-based methods developed within the theoretical framework of population genetics (Wright 1951; Nei 1972; Excoffier et al. 1992). While both methods rely on the covariance structures between all populations with no assumptions about the underlying evolutionary processes, the Population Graph method accounts for multiple relationships among populations using partial regression coefficients. Nineteen sub-populations (586 individuals) were analyzed using 19 neutral markers. Two main genetic groups of populations (group1 and group2) were detected and found to be connected to each other. These two groups were also detected based on the four VRN1 polymorphisms. The Population Graph topology is expected to strongly reflect the migration model, as shown by a simulation approach using N-island and one-dimensional stepping-stone models (Dyer 2007). The observed topology of the RDB population-variety differed from both the stepping-stone and the N-island model because a strong clustering was detected, highlighting a more complex migration system. This pattern seemed to be mostly shaped by human activities (in particular by seed diffusion practices). A similar pattern was encountered in natural populations of Sonoran Desert cactus (*Lophocereus schottii* L.) submitted to an historical vicariance (induced splitting of population, into discontinuous parts, by sea) (Dyer and Nason 2004).

In a study on a metapopulation of the seagrass *Poseidonia oceanica* in the Mediterranean basin, the authors highlighted the key role of a few populations as hubs for relaying gene flow (Rozenfeld et al. 2008). In the RDB case, five populations contributed to the transition between the two genetic groups and might play an analogous role. Yet, we should be cautious in the comparison because Rozenfeld et al. (2008) used a different network theory-based approach. In our study, the three populations from group2 (JAS04, JOP06, JFB05) were composed of haplotypes from classes II, III, or IV. As haplotypes from class II were very close to haplotypes from the class I, almost all alleles were shared between both classes, which could explain their position in the Population Graph (Fig. 3B). Except for one individual found in JFB03, there was thus no evidence that group2 received specific haplotypes or alleles from group1. Two populations of group1 (ALB03A and ALB06B) showed one specific allele from class III that explained their boundary position in the Population Graph. This shared allele could be the footprint of an ancestral common population rather than recent gene flow between the two groups of populations. With recent gene flows, we would expect a higher frequency of haplotypes intermediate between the two groups.

Intra-population genetic structure was studied through the haplotype spanning network. Indeed, defining the haplotype approach was relevant because as bread wheat is mainly a self-pollinated species [5–10% outcrossing (Enjalbert et al. 1998; Enjalbert and David 2000)] recombination is not expected to be frequent. Thus, pairwise linkage disequilibrium estimated for each pair of loci over all the 19 populations was significant for more than 80% of the cases. Haplotype clustering revealed 29 OT, while these were not detected using STRUCTURE-like softwares. Thus, when we used the INSTRUCT software (Gao et al. 2007) on this dataset, it induced instability in assigning OT to the genetic groups and altered likelihood values for the different number of ancestral group assessed (data not shown). As a consequence, the criterion to choose the optimal number of groups did not show a strong and stable elbow. Haplotype clustering highlighted different population substructures ranging from homogeneous populations (composed of only one haplotype class) to composite populations (composed of up to three haplotype classes). In addition, the global genotype richness (polyclonality) level was 19.4%. Polyclonality has been previously observed in cassava (*Manihot esculenta* Crantz) landraces (Elias et al. 2000, 2001; Pujol et al. 2005a, b) with values between 29% and 55% associated with an excess of heterozygote genotypes (−0.94 < *F*_IS_ < −0.37). This was because of a complex system of agricultural management: volunteer plants recruited from soil seed banks often resulted from outcrosses. The most productive volunteer plants, in general largely heterozygous, are propagated by clonal reproduction. For this reason, heterozygotes occured at a high frequency. In bread wheat, rare spontaneous cross-pollination can also occur, which could increase the heterozygosity. However, after successive generations of self-pollination, heterozygosity decreases. Thus, self-pollination in heterogeneous populations can lead to the maintenance of polyclonal or composite populations with a low level of heterozygotes, as has been shown in natural population of *Medicago truncatula* (Siol et al. 2008).

Following the practices of the different actors (farmers and genebank curators) have been divided into two distinct processes, one acting at the overall scale of the system, that is, seed diffusions, and the other acting locally, at the farm level, that is, reproduction of the seed lot, which is largely dependent on agronomic practices.

### Impact of the seed diffusion network on the genetic structure

As far as we know, this is the first interdisciplinary ethnobotanic and genetic study conducted at the level of a single population-variety. Previous studies have pointed out that seeds have such a symbolic importance for farmers. In most cases, farmers explain that they have been maintaining the same variety for a long time, even if they occasionally substitute entirely or mix their own seed with seed from external sources (Louette et al. 1997; Smale et al. 1999; Badstue et al. 2007), actions which would affect the genetic make-up of populations. Contrary to these situations, the genetic structure found in our study was highly consistent with the SDRNs obtained through interviews: within-SDRN cGD was significantly lower than between-SDRN cGD. Consistence between the rules described as structuring social networks of seed exchange between farmers communities and the genetic structure of manioc (*Manihot esculenta* Crantz) was also recently described in Gabon (Delêtre et al. 2011). In general, several cycles of reproduction are conducted between two events of seed diffusion. Recycling seeds from one’s own harvest is the backbone of local seed supply (Perales et al. 2003; Carpenter 2005; Delaunay et al. 2008). This is also what we observed in this network of actors. On average, the 19 populations sampled in this study had been grown 5.7 generations in the same farm since the previous diffusion event. In comparison, populations were grown from 4.1 to 15 generations in farmer communities in Ethiopia (McGuire 2007). In other words, in our study, 89% of the seed source comes from the previous harvest of the same farmer. This value is similar to those observed in local farming contexts [80% in farmer communities growing sorghum in Burkina Faso (Delaunay et al. 2008), 53% in farmer communities growing maize in Mexico (Louette et al. 1997)].

Seed diffusion can be considered as a colonization event in the metapopulation model with two basic mechanisms: the ‘migrant pool’ model and the ‘propagule pool’ model (Slatkin 1977). In the seed diffusion process described here, colonization events mainly correspond to the propagule model with the exception of one seed sample (JOP06), which came from seed mixtures (following the migrant model). Even though strong differentiation among subpopulations is expected because of strong founder effects in the propagule model of colonization (Whitlock and McCauley 1990), the fact that we found no evidence of connection between the two SDRNs might indicate that two independent founding effects have occurred in the past. In addition, as bread wheat is mainly a self-pollinated species, the differentiation might be increased by a family group founding effect (Ingvarsson and Giles 1999). This lack of evidence for connection was consistent with the high level of differentiation between the two connected components (SDRN1 and SDRN2: *F*_ST_ = 0.697). Furthermore, the fact that all the populations have been diffused suggested that populations might not yet have achieved equilibrium.

Thus, the genetic analysis provided new insights into the seed diffusion history and by extension into the associated social processes. Relying on information collected through the interviews, it was initially not possible to connect three populations (JEF06, FRP06, ALP05) to any SDRN although we collected seed circulation information back to the 1990s. With the molecular analyses of the population structure, it was possible to assign these three populations to the SDRN2, because they showed a pattern similar to that of SDRN2 populations. In addition, because two of them also presented a composite structure, we thought that the property of composite population was relatively old in the history of the RDB population-variety. Because JEF06 was not a composite population and showed no trace of alleles from haplotype class II while showing several satellite haplotypes from class III, JEF probably received a seed lot from a RDB population before the composite pattern occurred in SDRN2. We also showed that haplotypes at low frequency were shared by different populations of the SDRN2 (Fig. 6). This result confirmed that these populations were connected by seed circulation. Although a farmer (JFB) from SDRN2 received his RDB population from a unique source (ARC) (Fig. 2), we detected that his oldest RDB population (JFB03) was composed of individuals sharing three classes of haplotypes, including one belonging to class I. This is an argument for a complex ancestral population-variety composed of three main haplotype classes (I–III). However, this hypothesis needs to be considered carefully because only one individual was observed to come from haplotype class I. Furthermore, we showed that only a few specific alleles were shared between both SDRNs. An alternative hypothesis could be that two distinct cryptic varieties with almost the same phenotypic traits are being maintained independently in these two SDRNs.

### Impact of human local practices on the genetic structure

We showed that, on average, the genetic diversity observed in SDRN1 was significantly lower than that in SDRN2. According to the information collected during the interviews, populations from SDRN1 (Fig. 2, in blue) come from the formal seed sector. The initial donor of the SDRN1 populations was a breeder. Thus, these populations were initially subjected to a strong homogenizing pressure to follow the distinction, uniformity, and stability (DUS) criteria of the formal system. Consequently, the CLM genebank sample (CLM03) obtained from this source showed a much lower genetic diversity than most of the other samples. The trend for genebank accessions to have lower genetic diversity than *in situ* collection was also highlighted in several papers (see Negri et al. 2009 for a review). In contrast to the populations of SDRN1, the populations of SDRN2 have always been grown on farm without the DUS constraints and diversified agricultural practices among farms, so they were subjected to less homogenization.

Demographic size of crop populations is generally highly variable (Rice et al. 1998). In this context, population size could play an important role in the evolution of populations depending upon the seed quantity obtained after the diffusion event and/or the seed quantity recycled. Generally, actors who practice variety conservation grow their populations on small plots (a few m^2^), in contrast to others who follow multiplication, isolation, or production practices (field surfaces from 10 to several thousand m^2^). Genetic drift, particularly in diversified populations with a small demographic size, might reduce the genetic diversity and increase the genetic load. This situation could account for some patterns observed in SDRN1, because five populations out of seven were grown in small plots. However, as mentioned in the previous paragraph, the overall low level of genetic diversity found in SDRN1 could be explained by the historical conservative practices of the formal system. Using the temporal variation of allele frequencies between the two samples available at the farm BER resulted in an infinite estimate of effective size, *N*_e_, because allelic frequency variation was too low. This was associated with a low variation in terms of haplotype composition of the population between 2003 and 2006 which is consistent with the conservative practices used by BER. Except for JFB05 and JOP06, which followed cultural practices best described as selection, populations in SDRN2 seemed to have larger size than populations from SDRN1. Estimated *N*_e_ based on the JFB03 and JFB06 populations, within SDRN2, was of the same order of magnitude of bread wheat populations grown under dynamic management experiment [104.5 in this study compared with 123.0 after 10 generations of evolution in Goldringer et al. (2001)], while within-population genetic diversity was relatively high in these populations (0.32 and 0.31, respectively, for 2003 and 2006). This trend might be amplified when there was occasional past or recent mixture with other varieties (ARC80 and JOP06 respectively).

Migration is one of the evolutionary forces that could significantly influence the differentiation within the system. In the case of an open-pollinated species such as maize, pollen-mediated gene flow is important and generally leads to a low level of genetic differentiation, though farmers’ selection on ear type induces stronger phenotypic differentiation among landraces (Pressoir and Berthaud 2003). Because phenotypes are quite distinct between varieties and because wheat is a self-pollinated species, uncontrolled migration among populations is expected to be rare. However, the composite property of some populations of SDRN2 (mainly haplotype classes II and III) and the higher number of haplotypes observed in class III indicated that migration might have occurred in the past with individuals of haplotype class II that migrated into populations of haplotype class III. In addition, we know that haplotype class II is genetically very similar to class I, thus possibly indicating a common ancestral origin. While this is only the structure of the neutral genetic diversity, if a convergent phenotype was also to be observed between the different haplotype classes that could explain why farmers continue to grow these different populations under the same name RDB, a detailed phenotyping of these different haplotype classes would be helpful to confirm this point. The low outcrossing rate found in wheat [5–10% (Enjalbert et al. 1998; Enjalbert and David 2000)] is consistent with finding some recombinant individuals. This was observed in CLM04 and FRP06. Present at low frequencies, this phenomenon illustrates contact with other varieties. This is consistent with two identified practices: as already mentioned, some farmers have grown their RDB populations in mixture with other varieties, while other farmers maintain their populations in collections and grow them in small plots close together that could result in mixtures or outcrosses at different steps of the reproduction process.

Genetic differentiation (pairwise *F*_ST_) measured in neutral regions was highly correlated with genetic differentiation measured in VRN-1 genes involved in flowering time (adaptive trait) (Fig. 4). Divergent selection between wheat populations grown for several generations in contrasted sites would have led to specific patterns such as higher *F*_ST_ at genes under selection compared with *F*_ST_ at neutral markers (Vitalis et al. 2001; Rhoné et al. 2010). Thus, the structure of genetic diversity observed seems more influenced by actors’ practices rather than by the short-term environmental conditions where populations have been grown. Different types of selection can be described. The first is negative selection performed by farmers or genebank curators when they remove off-type plants that appeared spontaneously in the population in the field. These practices could explain the low rate of OT in the dataset. The second selection is positive: for example, the ear-based selection for the RDB ear type [red awnless (JOP06)]. The farmer explained that he received a mixture of different wheat varieties including RDB. He thus decided to select a few RDB ears type to initiate a new cycle of multiplication as a pure variety. This selected population showed low genetic diversity (unbiased *H*_e_ = 0.008) with only one class of haplotype detected (class II). Finally, there was another case of positive selection when in 2001, one farmer (JFB) made a selection of a new derived ear type (red awned) which appeared spontaneously in his RDB population. He further grew the progeny as a separate population, which he named ‘Rouge du Roc’. This process corresponds to the creation of a new population-variety related to RDB. In 2003, he gave a sample to CLM.

## Conclusion

This article investigated how human activities shape genetic diversity of crops at the variety level. We showed that the network of actors involved in the RDB cultivation or conservation strongly influenced the population-variety structure and maintained it under a nonequilibrium state. Using a metapopulation genetic framework helped us to identify two processes that led to coexistence of two cryptic genetic groups: (i) at the global scale, the combined analysis between the seed diffusion dynamics and the genotyping of RDB populations highlighted two distinct seed diffusion pathways which appeared to be strongly consistent with the genetic structure of this population-variety, (ii) cultural practice diversity affected the local scale (different population sizes, selection, migration…), leading to the maintenance of contrasting populations with a large range of diversity from fixed populations to composite populations.

From a genetic resources perspective, these results give convincing arguments to the stakeholders involved in genetic resource management for collecting critical information about seed circulation and cultural practices in the context of on-farm conservation of cultivated diversity. Here, we showed that on-farm conservation has the particular characteristic of maintaining intra-varietal genetic diversity. This leads us to emphasize the need to foster collaboration among partners from *ex situ* and *in situ* conservation to conserve crop genetic diversity at the different levels.

## Data archiving statement

Data deposited in the Dryad repository: doi: 10.5061/dryad.2fv7033c.

## Supporting Information

**Figure S1.** The haplotypic network of RDB based on 19 SSR.
