# Supplementary material for: On-farm dynamic management of genetic diversity: the impact of seed diffusions and seed saving practices on a population-variety of bread wheat
Source: Evol Appl. 2012 Dec;5(8):779–95. doi: 10.1111/j.1752-4571.2012.00257.x (PMC3552397; doi:10.1111/j.1752-4571.2012.00257.x)
Supplement: Supplementary file 1 [file eva0005-0779-SD1.doc]

**Figure S1.** The haplotypic network of RDB based on 19 SSR. Each node corresponds to one of the 119 distinct haplotypes contained among the 586 genotyped individuals. Two haplotypes are connected whether they differ by only one difference. Size of the node is proportional to the occurrence of this haplotype in the whole dataset using a logarithmic scale.
